# Serum matrix metalloproteinase-9 levels and enzymatic activity in patients with anti-glomerular basement membrane disease

**DOI:** 10.3389/fmed.2025.1668791

**Published:** 2025-11-18

**Authors:** Huang Kuang, Hong-shan Wan, Qiu-hua Gu, Xiao-yu Jia, Zhao Cui, Ming-hui Zhao

**Affiliations:** 1 Renal Division, Peking University First Hospital, Beijing, China; 2 Institute of Nephrology, Peking University, Beijing, China; 3 Key Laboratory of Renal Disease, Ministry of Health of China, Beijing, China; 4 Key Laboratory of CKD Prevention and Treatment, Ministry of Education of China, Beijing, China; 5 Department of Nephrology, Tianjin Medical University General Hospital, Tianjin, China

**Keywords:** anti-glomerular basement membrane disease, serum matrix metalloproteinase-9 (MMP-9), goodpasture antigen, clinical-pathological features, α3(IV)NC1

## Abstract

**Background:**

Anti-glomerular basement membrane (anti-GBM) disease is an autoimmune disorder with autoantibodies against GBM component, non-collagenous domain of the α3 chain of type IV collagen, α3(IV)NC1. Matrix metalloproteinase-9 (MMP-9) is an endogenous enzyme for the degradation of GBM to produce α3(IV)NC1. The current study was aimed to investigate the serum levels and enzymatic activity of MMP-9 and their clinical significance in patients with anti-GBM disease.

**Methods:**

MMP-9 serum levels and enzymatic activity were measured by enzyme-linked immunosorbent assay in 77 patients with anti-GBM disease and 20 healthy individuals. The association of MMP-9 levels and enzymatic activity with clinical-pathological features of patients was analyzed.

**Results:**

MMP-9 levels and enzymatic activity were elevated in 53.2% (41/77) and 61.0% (47/77) of patients with anti-GBM disease, and in 0% (0/20) and 10% (2/20) of healthy individuals (*P* < 0.001, *P* < 0.001, respectively). MMP-9 enzymatic activity was negatively correlated with serum creatinine (*r* = –0.247, *P* = 0.030) and positively correlated with eGFR (*r* = 0.232, *P* = 0.043). It was higher in the patients with prodromal infection [512.8 (286.0–682.8) vs. 307.2 (203.2–487.0) ng/mL, *P* = 0.037]. Patients with combined ANCA positivity had a significantly weaker MMP-9 enzymatic activity than that of patients with only positive anti-GBM antibodies [241.0 (192.3–431.9) vs. 444.5 (255.2–659.5) ng/mL, *P* = 0.035)]. Univariate regression analysis suggested that neither MMP-9 levels nor enzymatic activity was a risk factor for kidney outcome in patients with anti-GBM disease.

**Conclusion:**

The serum MMP-9 levels and enzymatic activity were elevated in patients with anti-GBM disease. The association between MMP-9 activity and clinical-pathological features indicated that MMP-9 might play different roles in disease development.

## Introduction

Anti-glomerular basement membrane (anti-GBM) disease is one of the classical autoimmune diseases, characterized by the development of circulating anti-GBM antibodies and deposition on kidneys and/or lungs, leading to rapidly progressive glomerulonephritis and/or lung hemorrhage (1). The main autoantigen of anti-GBM disease was identified as the non-collagenous domain of α3 chain of type IV collagen [α3(IV)NC1] (2, 3), with two conformational epitopes E_*A*_ (17–31) and E_*B*_ (127–141) on it (4). Both anti-GBM antibodies and T cell-mediated autoimmunity contribute to the pathogenesis of anti-GBM disease (5–8).

Matrix metalloproteinase-9 (MMP-9) is one of the family of zinc-dependent endopeptidases with collagenase activity (9–11). Type IV collagen is a major substrate for MMP-9 and one of its cleavage products is α3(IV)NC1, also known as tumstatin (10–13). Whether abnormal level of MMP-9 degrades type IV collagen to produce α3(IV)NC1 and thus contributes to the breakdown of immune tolerance in anti-GBM disease remains uncertain. MMP-9 is synthesized and released as inactive pro-MMPs, activated by plasmin or other MMPs, and inhibited by tissue inhibitors of matrix metalloproteinases (14). In normal conditions, MMP-9 mainly participates organ development and matrix degradation (9). In human diseases, however, elevated MMP-9 has been reported in many autoimmune diseases, such as rheumatoid arthritis (15, 16), multiple sclerosis (17, 18), and kidney diseases (19–24). However, there is a controversy regarding the role of MMP-9 in anti-GBM disease. Lelongt et al. found that the deficiency of MMP-9 *in vivo* aggregated the progression of anti-GBM nephritis as the ability of MMP-9 to cleave fibrin was an essential mediator of kidney injuries (25, 26). Conversely, Kluger et al. demonstrated a proinflammatory role of MMP-9 with the evidence that a reduced infiltration of proinflammatory macrophages in kidneys and an ameliorated disease progression were observed in MMP-9 deficient animal models of anti-GBM nephritis (27). The mRNA expression of *MMP-9* in the glomerulus was also increased during the development of anti-GBM nephritis in WKY rats (28). Together, these findings suggest that MMP-9 may play an important role in both fibrosis and inflammation during the course of anti-GBM nephritis.

However, the role of MMP-9 in patients with anti-GBM disease remains poor understood. In the present study, we measured serum MMP-9 level and activity in patients with anti-GBM disease. Their associations with clinical and pathological data were further analyzed, aiming to identify their clinical significance.

## Materials and methods

### Patients and sera collection

Seventy-seven consecutive Chinese patients with anti-GBM disease admitted to Peking University First Hospital from June 2010 to December 2016 were retrospectively reviewed. The diagnosis of anti-GBM disease was made by the presence of circulating anti-GBM antibodies and/or linear immunoglobulin G (IgG) deposits along the GBM under direct immunofluorescence on kidney biopsies. One patient had negative circulating anti-GBM antibodies detected by enzyme-linked immunosorbent assay (ELISA) and linear IgG deposits along GBM confirmed his diagnosis of anti-GBM disease. Clinical data were collected from medical records. Prodromal infection was described in our previous study and defined as infections before the onset of anti-GBM disease (29). Prodromal infection occurred at least one month prior to disease onset and the collection of serum samples. The kidney fibrosis was characterized by area of collagen deposition on dual periodic acid–Schiff (PAS) and Masson staining and semi-quantified by professional pathologists. In detail, “0” was characterized by no fibrosis area, “1” was focal fibrosis (< 25% area), “2” was multifocal fibrosis (25–75% area), and “4” was diffuse fibrosis (> 75% area).

Sera from these enrolled patients were collected before immunosuppressive treatment and plasma exchange, and preserved at –40°C until experiments. Sera from twenty healthy individuals were collected as healthy controls (HCs). This study complied with the *Declaration of Helsinki* and written ethics was approved by the Ethics Committee of Peking University First Hospital. Informed consent was obtained from all individuals.

### Detection of anti-GBM antibodies and anti-neutrophil cytoplasmic antibodies

Detection of anti-GBM antibodies was performed using commercial ELISA kits (EUROIMMUN, Lübeck, Germany; positive cut-off value was set as > 20 RU/mL) with purified bovine α3(IV)NC1 as the solid-phase antigen, and confirmed antibodies specificity using ELISA against recombinant human α3(IV)NC1. The detection of ANCA was performed using indirect immunofluorescence (EUROIMMUN) with ethanol-fixed human neutrophils. Antigen-specific ELISA (EUROIMMUN) was performed against purified myeloperoxidase and proteinase 3.

### Detection of MMP-9 levels in sera

We detected the serum MMP-9 levels in human subjects using the Quantikine Human MMP-9 Immunoassay (R&D Systems, Minneapolis, United States), according to the manufacturer’s protocol. In brief, 96-well polystyrene microplates were precoated with mouse monoclonal antibodies specific for human MMP-9. Gradient-diluted standards and sera with a dilution of 1:100 were added to each well respectively and incubated for 2 h at room temperature. After incubation and washing, horseradish peroxidase-conjugated polyclonal antibodies specific for human MMP-9 were added and incubated for 1 h at room temperature. After washing, substrate solution was added to each well and incubated for 30 min at room temperature in darkness. After that, stop solution was added and the absorbance was recorded using an ELISA reader (Bio-Rad Laboratories, Philadelphia, PA) at 450/570 nm. The MMP-9 levels of each sample were calculated using Curve expert 1.3 (Curve Expert Software, Chattanooga, TN).

### Detection of MMP-9 activity in sera

We detected the MMP-9 enzymatic activity in sera using Matrix Metalloproteinase-9 (MMP-9) Biotrak Activity Assay System (Amersham Pharmacia Biotech UK Limited, Little Chalfont, United Kingdom), according to the manufacturer’s protocol. In brief, 96-well polystyrene microplates were precoated with monoclonal antibodies specific for human MMP-9. Human pro-MMP-9 was used as a standard sample with different concentrations. Sera were added with a dilution of 1:40 and incubated at 4°C overnight. After washing, p-aminophenylmercuric acetate solution was added to activate pro-MMP-9 and incubated for 1.5 h at 37°C. Then, a detection reagent included modified murokinase was added and the plate was read at 405 nm to obtain OD value using an ELISA reader (Bio-Rad Laboratories, Philadelphia, PA). The plate was incubated at 37°C for 2 h and the absorbance at 405 nm was recorded again. MMP-9 activity was calculated as formula “OD_405_/2*1000” according to the manufacturer’s instructions.

### Treatments and outcomes

Plasma exchange (2–4 L) was performed daily or every other day up to 14 times or until circulating anti-GBM antibodies were undetectable. Human albumin (5%) was used as a replacement material. Fresh frozen plasma was used in patients with lung hemorrhage. Methylprednisolone pulse therapy (7–15 mg/kg per d, < 1 g/d) was given for 3 days, followed by prednisone (1 mg/kg per d, < 60 mg/d) with gradual tapering within 6–12 months. Oral cyclophosphamide (2–3 mg/kg/d) was given for 2–3 months. Follow-up data were obtained from patients’ medical records. The primary outcome was kidney survival. End-stage kidney disease (ESKD) was defined as dialysis dependence for > 3 months.

### Statistical analysis

Data were presented as *n* (percentage), mean ± standard deviation (SD), or median (P25, P75). All the statistical analyses were performed using SPSS statistical software package, version 26.0 (SPSS Inc., Chicago, IL, United States). Differences of quantitative parameters were assessed using the *t*-test for data that were normally distributed or non-parametric test for data that were not normally distributed. Differences of qualitative data were compared using the chi-square test/Fisher’s exact test. For correlation analysis of continuous data, Spearman correlation analysis was used as both MMP-9 serum levels and enzymatic activity were not normally distributed. For analyzing differences of MMP-9 serum levels and enzymatic activity among categorical data, Mann-Whitney test was used. The potential risk factors for ESKD were analyzed using a logistic regression model. All statistical analyses were two-tailed and a *P-*value < 0.05 was considered statistically significant.

## Results

### Demographic and clinical characteristics of patients with anti-GBM disease

Seventy-seven patients with anti-GBM disease were enrolled in this study. Their mean age was 51.5 ± 16.2 years. Among them, 40 were male, and 37 were female. 25 (32.5%) patients occurred prodromal infection before disease onset. The mean level of anti-GBM antibodies was 148.4 ± 60.8 RU/mL. 14 (18.2%) patients were combined with ANCA positivity. Among them, kidney biopsies were performed in 33 patients, and the mean crescent percentage was 71.4 ± 32.9%. The major clinical parameters were listed in Table 1.

**TABLE 1:** The demographic and clinical features of patients with anti-GBM disease.

Parameters	*n* = 77
Male/female	40/37
Age (years)	51.5 ± 16.2
Smoking (*n*,%)	31 (40.3)
Hydrocarbon exposure (*n*,%)	11 (14.3)
Prodromal infection (*n*,%)	25 (32.5)
Hemoptysis (*n*,%)	20 (26.0)
Gross hematuria (*n*,%)	21 (27.3)
Oliguria/anuria (*n*,%)	31 (40.3)
Urinary protein (g/24 h)	2.1 (0.7–5.6)
Nephrotic syndrome (*n*,%)	9 (11.7)
Hemoglobin (g/L)	92.4 ± 19.9
Albumin (g/L)	30.3 ± 5.5
Serum creatinine (mmol/L)	758.9 (465.0–1124.0)
ANCA (*n*,%)	14 (18.2)
Serum anti-GBM antibodies (RU/mL)	148.4 ± 60.8
Crescents (%)	71.4 ± 32.9
MMP-9 serum levels (ng/mL)	449.9 (303.3–878.9)
MMP-9 enzymatic activity (ng/mL)	355.7 (212.8–588.3)
Plasma exchange (*n*,%)	66 (85.7)

GBM, glomerular basement membrane; ANCA, anti-neutrophil cytoplasmic antibodies; MMP-9, matrix metallopreoteinases-9.

### Serum MMP-9 levels and enzymatic activity were elevated in patients with anti-GBM disease

**FIGURE 1:**
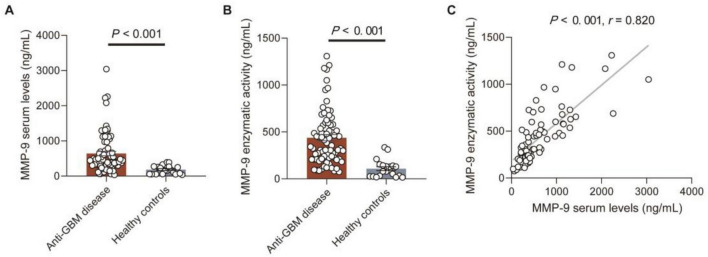
MMP-9 serum levels and enzymatic activity in patients with anti-GBM disease and healthy controls. **(A,B)** MMP-9 serum levels and enzymatic activity. Data are expressed as mean ± SEM; statistical significance was determined by Mann-Whitney test. **(C)** Correlation between serum MMP-9 levels and MMP-9 enzymatic activity. The *P-*value was determined by linear regression analysis and Spearman’s correlation coefficient (*r*) are indicated on the graph.

The serum MMP-9 levels and enzymatic activity in patients with anti-GBM disease and HCs were detected, respectively by commercial ELISA kits. The serum MMP-9 levels and enzymatic activity in patients with anti-GBM disease were significantly higher than those of HCs [449.9 (303.3–878.9) vs. 167.6 (57.6–273.3) ng/mL, *P* < 0.001; 355.7 (212.8–588.3) vs. 87.0 (28.5–142.4) ng/mL, *P* < 0.001, respectively] (Figures 1A,B). Using the mean + 2SD of serum MMP-9 levels and enzymatic activity from HCs as the cut-off values, MMP-9 levels and enzymatic activity were elevated in 53.2% (41/77) and 61.0% (47/77) of patients with anti-GBM disease, and in 0% (0/20) and 10% (2/20) of HCs (*P* < 0.001, *P* < 0.001, respectively). In patients with anti-GBM disease, serum MMP-9 levels had a strong correlation with the MMP-9 enzymatic activity (*r* = 0.820, *P* < 0.001) (Figure 1C).

### Correlations between MMP-9 levels and enzymatic activity and clinical-pathological data in patients with anti-GBM disease

**FIGURE 2:**
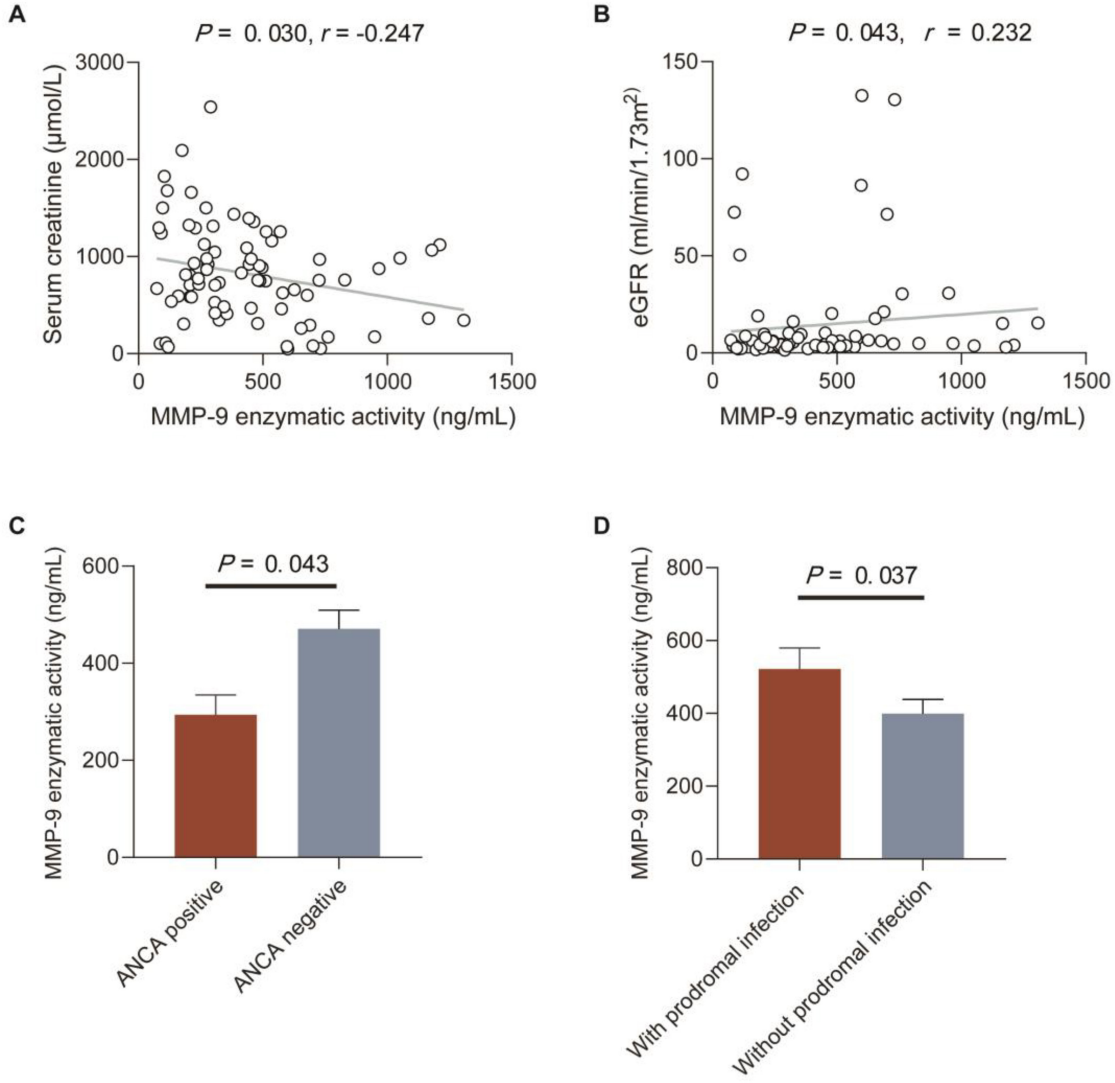
Correlations between MMP-9 enzymatic activity and clinical-pathological data in patients with anti-GBM disease. **(A,B)** Correlations between MMP-9 enzymatic activity and serum creatinine and eGFR. The *P-*value was determined by linear regression analysis and Spearman’s correlation coefficient (*r*) are indicated on the graph. **(C)** MMP-9 enzymatic activity in patients with or without ANCA. **(D)** MMP-9 enzymatic activity in patients with or without prodromal infection. Data are expressed as mean ± SEM in **(C,D)**; statistical significance was determined by Mann-Whitney test **(C,D)**.

Correlations between MMP-9 levels and enzymatic activity and clinical-pathological data in patients with anti-GBM disease were analyzed, respectively (Tables 2, 3). We found serum MMP-9 enzymatic activity correlated negatively with serum creatinine (*r* = –0.247, *P* = 0.030), but MMP-9 levels did not (*r* = –0.209, *P* = 0.069) (Figure 2A). MMP-9 enzymatic activity was correlated positively with estimated glomerular filtration rate (eGFR) (*r* = 0.232, *P* = 0.043), rather than MMP-9 levels (*r* = 0.212, *P* = 0.064) (Figure 2B). Patients with combined ANCA positivity had a significantly weaker MMP-9 enzymatic activity than that of patients only positive for anti-GBM antibodies [241.0 (192.3–431.9) vs. 444.5 (255.2–659.5) ng/mL, *P* = 0.043)] (Figure 2C). We also found MMP-9 enzymatic activity was higher in the patients with prodromal infection [512.8 (286.0–682.8) vs. 307.2 (203.2–487.0) ng/mL, *P* = 0.037] (Figure 2D). No correlation between MMP-9 levels/enzymatic activity and levels of anti-GBM antibodies or crescent percentage was found (*P* > 0.05).

**TABLE 2:** Correlations between MMP-9 levels and enzymatic activity and clinical-pathological data of patients with anti-GBM disease (categorical variable).

	MMP-9 enzymatic activity (ng/mL)		*P*-value	MMP-9 levels (ng/mL)		*P*-value
Male/female	303.2 (200.9–511.7)	434.9 (279.2–653.5)	0.078	457.9 (285.3–782.7)	449.9 (314.1–790.6)	0.737
Smoking (Y/N)	452.9 (220.3–524.0)	349.6 (215.9–598.9)	0.967	548.2 (302.6–895.1)	407.0 (308.2–766.9)	0.506
Hydrocarbon exposure (Y/N)	324.1 (161.6–482.1)	369.8 (227.9–592.5)	0.399	443.8 (205.6–803.5)	461.0 (308.2–775.1)	0.600
Prodromal infection (Y/N)	512.8 (286.0–682.8)	307.2 (203.2–487.0)	**0.037**	623.5 (304.0–1144.2)	418.2 (311.2–659.7)	0.162
Hemoptysis (Y/N)	360.3 (220.9–479.3)	355.6 (213.3–599.6)	0.436	404.0 (302.6–721.2)	550.9 (320.5–990.7)	0.493
Gross hematuria (Y/N)	569.7 (264.2–948.5)	333.9 (213.1–500.4)	0.078	395.5 (303.7–645.5)	658.7 (377.4–1127.5)	0.091
Oligoanuria (Y/N)	463.4 (273.2–571.9)	324.1 (207.3–592.5)	0.192	623.5 (320.9–953.1)	435.2 (302.6–652.8)	0.321
Nephrotic syndrome (Y/N)	306.2 (264.2–355.6)	424.4 (211.0–584.1)	0.646	465.9 (311.6–834.7)	352.4 (212.8–473.5)	0.485
ANCA positivity (Y/N)	241.0 (192.3–431.9)	444.5 (255.2–659.5)	**0.043**	374.8 (317.9–596.5)	482.0 (303.3–977.8)	0.319
Initial need for RRT (Y/N)	315.9 (224.9–507.7)	452.9 (210.3–670.8)	0.367	415.3 (303.0–658.5)	482.0 (314.4–1126.5)	0.232
Plasma exchange (Y/N)	324.1 (215.9–573.1)	485.8 (298.1–650.6)	0.476	446.9 (304.6–923.0)	482.0 (315.9–695.8)	0.839

Statistically significant differences were reported with a bold *P*-value. Y/N, Yes/No. ANCA, anti-neutrophil cytoplasmic antibodies; RRT, renal replacement therapy.

**TABLE 3:** Correlations between MMP-9 levels and enzymatic activity and clinical-pathological data of patients with anti-GBM disease (continuous variable).

	MMP-9 enzymatic activity (ng/mL)		MMP-9 levels (ng/mL)	
	*R*	*P*	*r*	*P*
Age (years)	–0.005	0.968	0.007	0.954
Hemoglobin (g/L)	0.162	0.159	0.138	0.233
Albumin (g/L)	0.016	0.890	0.016	0.088
Serum creatinine (mg/dL)	–0.247	**0.030**	–0.209	0.069
eGFR (mL/min per 1.73 m^2^)	0.232	**0.043**	0.212	0.065
Serum anti-GBM antibodies (RU/mL)	0.109	0.347	–0.018	0.877
Crescents (%)	0.074	0.682	0.138	0.444

Statistically significant differences were reported with a bold *P*-value. GBM, glomerular basement membrane; ANCA, anti-neutrophil cytoplasmic antibodies; MMP-9, matrix metallopreoteinases-9; eGFR, estimated glomerular filtration rate.

### Treatment responses and kidney outcomes

Of the 77 patients with anti-GBM disease, 66 (85.7%) patients received plasma exchange combined with immunosuppressive treatment. After the treatment, 52 (67.5%) patients went to ESKD. Univariate logistic regression analysis showed that the risk factors affecting kidney outcome were oliguria/anuria [odds ratio (OR) = 5.670; 95% confidence interval (CI): 1.708–18.819; *P* = 0.005], serum creatinine (OR = 1.005; 95%CI: 1.003–1.007; *P* < 0.001), levels of anti-GBM antibodies (OR = 1.009; 95%CI: 1.001–1.017; *P* = 0.027), and crescent percentage (OR = 1.075; 95%CI: 1.021–1.131; *P* = 0.006) (Table 4). MMP-9 enzymatic activity was not a risk factor for ESKD in patients with anti-GBM disease (OR = 1.000; 95% CI: 0.998–1.001; *P* = 0.660). The level of MMP-9 was not, either (OR = 1.000; 95%CI: 0.999–1.001; *P* = 0.971). Previous studies suggested that crescents > 50%, serum creatinine > 4.7 mg/dL, and initial need for renal replacement therapy (RRT) were associated with worse kidney outcome (30), and these findings were also validated in our current study (*P* < 0.05) (Table 4). When we conducted the multivariate logistic regression by adding these variables to analyze their influences toward MMP9 enzymatic activity associated with kidney outcome, only initial need for RRT was strong prognostic factor for ESKD (Supplementary Table 1).

**TABLE 4:** Factors affecting kidney outcome of patients with anti-GBM disease.

	OR	95%CI	*P*-value
Male/female	1.605	0.614–4.194	0.335
Age (years)	0.974	0.944–1.006	0.110
Prodromal infection (n,%)	1.644	0.605–4.470	0.330
Hemoptysis (n,%)	3.562	0.934–13.579	0.063
Gross hematuria (n,%)	2.550	0.756–8.604	0.131
Oliguria/anuria (n,%)	5.670	1.708–18.819	**0.005**
Nephrotic syndrome (n,%)	0.333	0.081–1.371	0.128
Albumin (g/L)	0.984	0.900–1.076	0.725
Serum creatinine (mg/dL)	1.005	1.003–1.007	**< 0.001**
Serum creatinine > 4.7 mg/dL	35.3	9.070–185.0	**< 0.001**
ANCA (n,%)	2.017	0.508–8.004	0.319
Serum anti-GBM antibodies (RU/mL)	1.009	1.001–1.017	**0.027**
Crescents (%)	1.075	1.021–1.131	**0.006**
Crescents > 50%	16.3	2.250–338.0	**0.004**
Initial need for RRT	57.0	13.4–406.0	**< 0.001**
The level of MMP-9 (ng/mL)	1.000	0.999–1.001	0.971
The activity of MMP-9 (ng/mL)	1.000	0.998–1.001	0.660

Statistically significant differences were reported with a bold *P*-value. GBM, glomerular basement membrane; ANCA, anti-neutrophil cytoplasmic antibodies; RRT, renal replacement therapy; MMP-9, matrix metallopreoteinases-9; OR, odds ratio; CI, confidence interval.

## Discussion

The circulating levels and enzymatic activity of MMP-9 in patients with anti-GBM disease were first detected in this study. Type IV collagen is one substrate of MMP-9 and the cleavage product tumstatin could suppress angiogenesis via αVβ3 integrin under physiological conditions (12, 13). However, the concentration of tumstatin will be increased and released into the circulation when the level or activity of MMP-9 is abnormal. Tumatatin is α3(IV)NC1, the main target antigen of anti-GBM antibodies. The special association of MMP-9 and type IV collagen inspires us to investigate the serum level and enzymatic activity of MMP-9 in patients with anti-GBM disease and their clinical significance.

In the study, we found that both the level and the enzymatic activity of MMP-9 in sera of 77 patients with anti-GBM disease were significantly higher than that of HCs. The level of circulating MMP-9 varies in patients with different kidney diseases, and even in the context of the same disease its level is controversial in several publications. For example, Bauvois et al. found that both patients with IgA nephropathy and membranous nephropathy exhibited a significant reduction in plasma MMP-9 (20). However, these findings were contradictory to evidence from Endo et al. who did not observe any statistically significant differences from those of HCs (19). There are also controversies about the level and enzymatic activity of MMP-9 in patients with systemic lupus erythematosus and diabetic kidney disease (22, 31–34). These findings imply that circulating MMP-9 may function differently in diverse kidney diseases.

In previous studies, several *in vivo* studies have investigated the role of MMP-9 in the pathogenesis of anti-GBM disease. In the accelerated model of nephrotoxic serum nephritis (NTN), Lelongt et al. found that kidney injuries were worse in *MMP-9*^–/–^ mice than that of wild-type mice and that the protective mechanism of MMP-9 against the disease progression may be attributed to the degradation of fibrin by MMP-9 (25). However, Kluger et al. further indicated that the severity of non-accelerated NTN was alleviated in *MMP-9*^–/–^ mice, with the possible mechanism that the reduction of infiltrating monocytes and macrophages within kidneys induced by MMP-9 (27). Possible reasons for the different roles of MMP-9 could be due to the different origins of MMP-9 or MMP-9 functions differently in two phages (heterologous and autologous) during NTN development. The proinflammatory effect of MMP-9 might come from leukocytes, while its proteolytic effect might be expressed by kidney resident cells. The majority of circulating MMP-9 are derived from leukocytes (35, 36). In the present study, we found the circulating MMP-9 was significantly higher than that of the HCs, and positively correlated with prodromal infection in the patients with anti-GBM disease. Due to the different types of infections, the number of leukocytes increases, which leads to an increased secretion of MMP-9. Both MMP-9 level and enzymatic activity did not have correlations with the crescent percentage and interstitial fibrosis, or inflammatory cells infiltration in kidneys, indicating that circulating MMP-9 might not affect the kidney injury. MMP-9 was a key mediator in the development of kidney fibrosis, whereas no correlation was found between MMP-9 activity/levels and renal fibrosis in our current study. We hypothesize that the rapid progressive nature of anti-GBM disease, which often leads to ESKD within weeks, creates a pathological context distinct from the chronic models (e.g., diabetic kidney disease, unilateral ureteral obstruction) where the pro-fibrotic role of MMP-9 is well-established (37–40). In these chronic settings, MMP-9 acts over months to years in a sustained cycle of extracellular matrix degradation and remodeling that drives fibrosis. In contrast, during fulminant injury of anti-GBM disease, the primary role of MMP-9 may be shifted toward acute processes such as inflammation chemotaxis, disruption of the glomerular basement membrane, and facilitation of crescent formation (27). The pro-fibrotic programs, which are characteristic of a later, reparative (albeit maladaptive) phase, may be temporally overshadowed or pre-empted by the rapidity of the destructive injury. The serum MMP-9 level and enzymatic activity negatively correlated with serum creatinine and ANCA positivity, which usually means severer nephritis. Meanwhile, there is a positive correlation between the serum MMP-9 enzymatic activity and eGFR. One study by Kuroda et al. found that the mRNA expression of *MMP-9* increased in the early stage of anti-GBM nephritis and decreased further along with disease progression (28). That might be the reason that our study did not find the MMP-9 level and enzymatic activity as risk factors for kidney outcome. The previous studies did not show significant correlations between serum MMP-9 and clinical data in other glomerular diseases, either (19, 20, 31, 32).

As MMP-9 has a major collagenase activity for type IV collagen, and the final product, tumstatin, is happened to be the target of anti-GBM antibodies. Previous studies found there were GBM fractions in human urine and blood (41). In our study, the MMP-9 level and activity increased in the circulation of patients with anti-GBM disease. Therefore, we speculated that the increased concentrations of MMP-9 will result in soluble tumstatin released into the circulation by degrading the type IV collagen of GBM. More importantly, both α3(IV)NC1-specific T cells and natural anti-GBM antibodies were present in the sera of healthy individuals (42, 43), and thus the exposure and expansion of α3(IV)NC1 might contribute to the breakdown of immune tolerance and trigger the autoimmunity leading to the development of anti-GBM disease (44). However, we did not find a correlation between MMP-9 and circulating anti-GBM antibodies. The possible reason might be that the amino acids essential for the antiangiogenic activity of tumstatin are different from the motif critical for the antigenicity of α3(IV)NC1 (45, 46). The concentration of circulating tumstatin in patients with anti-GBM disease needs further detection. It would also be better to test the effect of pharmacological inhibition of MMP-9 (e.g., doxycycline) (47) in animal models of anti-GBM disease.

## Conclusion

In conclusion, we found the circulating MMP-9 level and enzymatic activity were significantly elevated in patients with anti-GBM disease. The function of MMP-9 in the development of anti-GBM disease needs further investigations.

## Data Availability

The original contributions presented in the study are included in the article/Supplementary material, further inquiries can be directed to the corresponding author.
