# Supplementary material for: Serum matrix metalloproteinase-9 levels and enzymatic activity in patients with anti-glomerular basement membrane disease
Source: Front Med (Lausanne). 2025 Nov 18;12:1668791. doi: 10.3389/fmed.2025.1668791 (PMC12668966; doi:10.3389/fmed.2025.1668791)
Supplement: Supplementary file 1 [file Data_Sheet_1.pdf]

Supplemental Table 1. Multivariate Logistic regression for factors affecting kidney outcome of patients with anti-GBM disease.

Characteristic	N	OR[1]	95% CI[1]	p-value
Serum creatinine (by 4.7 mg/dL)	33	3.56	0.24, 99.0	0.4
Crescents (by 50%)	33	15.1	0.66, 810	0.089
Renal replacement therapy	33	20.0	1.13, 844	**0.041**
MMP9 activity	33	1.00	0.99, 1.00	0.3
MMP9 level	33	1.00	1.00, 1.01	0.3

[1]OR = Odds Ratio, CI = Confidence Interval
